# New Phosphine Oxides as High Performance Near- UV Type I Photoinitiators of Radical Polymerization

**DOI:** 10.3390/molecules25071671

**Published:** 2020-04-04

**Authors:** Céline Dietlin, Thanh Tam Trinh, Stéphane Schweizer, Bernadette Graff, Fabrice Morlet-Savary, Pierre-Antoine Noirot, Jacques Lalevée

**Affiliations:** 1 Université de Haute-Alsace, CNRS, IS2M UMR 7361, F-68100 Mulhouse, France; thanh-tam.trinh@uha.fr (T.T.T.); stephane.schweizer@yahoo.fr (S.S.); bernadette.graff@uha.fr (B.G.); fabrice.morlet-savary@uha.fr (F.M.-S.); 2 Université de Strasbourg, France; 3 Siegwerk Druckfarben AG & Co. KGaA, Alfred-Keller-Str. 55, 53721 Siegburg, Germany; Pierre-Antoine.Noirot@siegwerk.com

**Keywords:** photopolymerization, photoinitiators, LED, phosphine-oxide

## Abstract

Carbazole structures are of high interest in photopolymerization due to their enhanced light absorption properties in the near-UV or even visible ranges. Therefore, type I photoinitiators combining the carbazole chromophore to the well-established phosphine-oxides were proposed and studied in this article. The aim of this article was to propose type I photoinitiators that can be more reactive than benchmark phosphine oxides, which are among the more reactive type I photoinitiators for a UV or near-UV light emitting diodes (LED) irradiation. Two molecules were synthesized and their UV-visible light absorption properties as well as the quantum yields of photolysis and photopolymerization performances were measured. Remarkably, the associated absorption was enhanced in the 350–410 nm range compared to benchmark phosphine oxides, and one compound was found to be more reactive in photopolymerization than the commercial photoinitiator TPO-L for an irradiation at 395 nm.

Academic Editor: Jamal Uddin

## 1. Introduction

Photopolymerization is an important process of synthesis of polymers as it is mostly a green technology (low energy consumption, no use of solvent, reaction at low temperature). Therefore, it has applications in many industrial fields such as coatings, inks, adhesive optoelectronics, nanotechnologies, and 3D-printing [1,2,3,4,5,6,7,8,9,10]. Another unique property of photopolymerization is its safety, i.e., when the irradiation is turned off, radicals are no longer generated, in contrast to thermal initiators that keep decomposing. The use of light emitting diodes (LEDs) in the near-UV region clearly offers advantages compared to the use of a classical UV Hg-lamps (no use of Hg, long lifetime, no heat release) but needs to be adapted to the chemical system used to initiate the photopolymerization reaction [11,12,13]. 

In fact, the absorption of the photoinitiator (PI) must match the emission spectrum of the light source. Therefore, only a few commercially available type I and type II photoinitiators can be used with LEDs, ranging from 365 to 405 nm (mostly BAPO, TPO, and TPO-L for type I (cleavable) PI; ITX and CQ for type II PI (with an H-abstraction reaction on the co-initiator) [1,5]. Therefore, to promote the use of LEDs in the near-UV range, new photoinitiators must be developed.

The carbazole structure is very interesting in photopolymerization. Some derivatives were used as additives to boost the photopolymerization reaction (radical and cationic processes) [14]. More recently, other carbazole derivatives have been used as photoinitiators for LED polymerization [15] and showed that their good reactivity was mostly due to their high absorbance around 400 nm in two-component systems. Carbazoles have also been introduced on classical type II photoinitiators such as thioxanthone to enhance both the absorption properties [16,17,18] and their reactivity. Another advantage is to have one component type II PI with an intermolecular H abstraction between the thioxanthone moiety and the carbazole moiety of two molecules, limiting the use of amines (even if the reactivity is enhanced in the presence of amines, see [18]). For type I PI, only one article mentioned the efficiency of carbazole phosphine oxide [19] and only for two-photon induced polymerization (not upon LED light) without any data on one-photon initiation. Therefore, we decided to study type I carbazole derivative PIs that should present an enhanced absorption around 380–405 nm and still keep some bleaching properties characteristic of phosphine oxide derivatives. 

**Figure 1:**
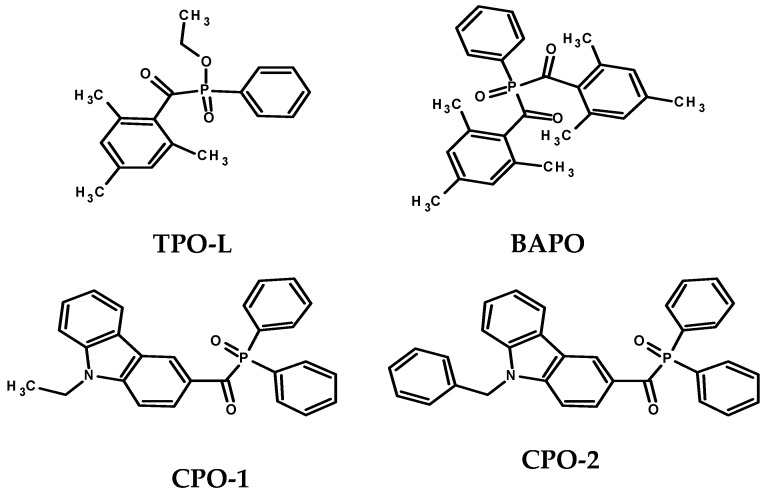
Chemical formula of the phosphine derivatives studied in this article.

Acyl phosphine oxides are very efficient type I PIs that have an n-π* absorption band located around 350–380 nm [1,5]. As the absorption band is broad, they still absorb light around 400 nm: The typical wavelengths of near-visible LEDs are 385 nm, 395 nm, and 405 nm. One way to improve their reactivity around 400 nm is to shift their absorption band toward 400 nm and/or enhance their absorption. This strategy has already been done by carefully adding a selected moiety on the acyl phosphine oxide structure, such as a benzophenone moiety in [20], an amine moiety in [21], or methyl and methoxy groups in [22]. Recent work with diacylphosphine oxides were performed in [23] and [24], with the modification of the moiety on the phosphorus atom in order to see its influence on water solubility and the reactivity parameters. However, the carbazole chromophore was not tested on acyl phosphine oxide derivatives for classical photopolymerization. That will be done in this article through a comparison of the reactivity of the proposed compounds (Figure 1) with a commercial type I photoinitiator. New PIs can allow for the reduction of the quantity of product needed to reach a good reactivity. This could be an asset from a toxicology point of view, to use as little compound as possible.

## 2. Results

**Scheme 1:**
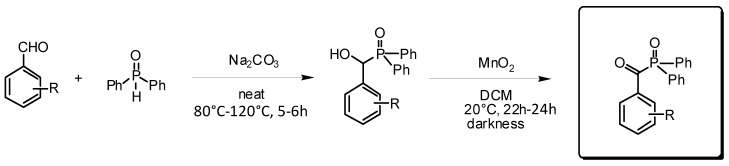
The two-step procedure used to synthesize new CPO in this paper.

The synthesis of the CPO derivatives was performed in a two-step reaction (Scheme 1) following the procedure presented in [19] (only minor adaptations in the use of solvent). Details can be found in the experimental part of the article, but the synthesis was straightforward. CPO-1 is the molecule already studied in [19] but only for 2-photon induced polymerization (not upon LED light) but CPO-2 is new and has never been studied. The experimental details are given below for their NMR characterization.

## 3. Discussion

**Figure 2:**
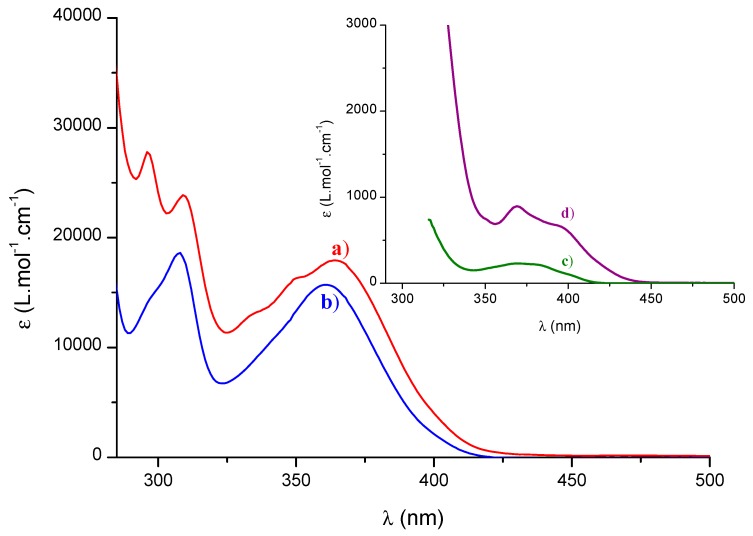
Molar extinction coefficient of a) CPO-1, b) CPO-2, c) TPO-L, and d) BAPO in acetonitrile.

**Figure 3:**
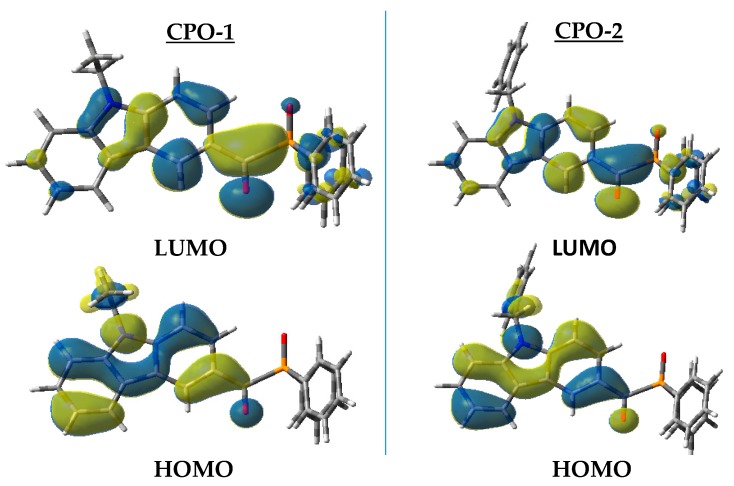
Molecular orbitals HOMO and LUMO in CPO-1 and CPO-2.

The absorption spectra of the CPO molecules are reported in Figure 2, showing that CPO-1 and CPO-2 exhibited similar absorption spectra in acetonitrile. More importantly, these compounds possessed molar extinction coefficients at least one order of magnitude higher than the that of the best type I commercially available PI (BAPO) at their maximum absorption (around 370 nm). Even at 395 nm, the molar coefficients of CPO-1 and CPO-2 were still higher than those of BAPO and TPO-L (Table 1). These results are also in agreement with the data obtained from molecular modeling (Table 1). The increase of the molar absorption comes from a delocalization of the highest occupied molecular orbital (HOMO) and the lowest unoccupied molecular orbital (LUMO) on the carbazole scaffold (Figure 3). A partial charge transfer was also observed in both moieties between the ground state and the excited state of the molecule. These data are in agreement with the expected improvement of the absorption properties with the use of a carbazole chromophore.

**Scheme 2:**
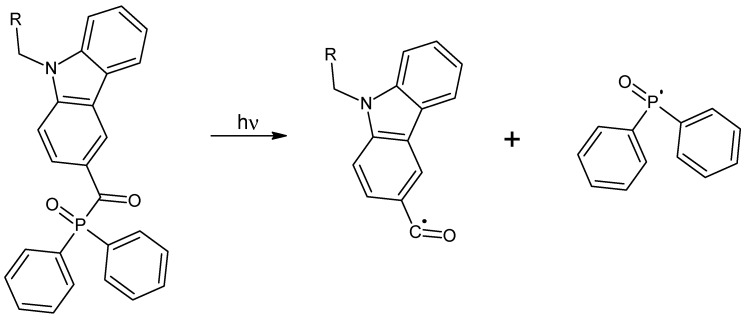
Expected cleavage reaction of CPO molecules by analogy with the reaction of TPO-L. For CPO-1, R = CH_3_, for CPO-2, R = Ph.

Then, the cleavage reaction was studied through the evaluation of the quantum yield of photolysis, i.e., the number of molecules that react for one photon absorbed. Other techniques to monitor the cleavage quantum yields are proposed in [25]. A C–P cleavage process occurs as the triplet state energies of CPO-1 and CPO-2 were calculated to be higher (62 kcal/mol) than the C–P bond dissociation energy (61 kcal/mol), suggesting a favorable cleavage process from this excited state. Such a behavior was found for other phosphine-oxides in [22]. The UV-vis spectra during photolysis showed a clear disappearance of the peak, in agreement with a C–P cleavage process. The quantum yields of photolysis of phosphine oxides such as BAPO and TPO-L are of medium size [5], below unity but still high enough to ensure a good reactivity of the compounds (UV photoinitiators are usually characterized by dissociation quantum yields > 0.2 [2]). For CPO-1 and CPO-2, the same C–P cleavage process was expected (Scheme 2) as the same bleaching to that of TPO-L or BAPO at the lowest energy transition was observed. The quantum yields of photolysis (ϕ) of CPO-1 and CPO-2 were close to that of BAPO, but slightly higher. Therefore, these molecules will cleave more easily than BAPO and TPO-L, leading to a faster phosphinoyl and carbonyl radical production (Scheme 2).

The Mulliken spin densities of the radicals produced after cleavage of the C–P bond in CPO-1 and CPO-2 were computed and values of 0.648 and 0.482 were found for the acyl radical and the phosphinoyl radical, respectively. The spin densities for TPO-L and BAPO were 0.641 for the acyl radical and 0.509 and 0.426 for the phosphinoyl radicals, respectively. These results show that the acyl radical produced for CPO-1 and CPO-2 should have the same reactivity as the ones produced by TPO-L and BAPO. For the phosphinoyl radical, the reactivity order should be TPO-L > CPO-1 = CPO-2 > BAPO, based on molecular modeling results of spin localization. Therefore, the introduction of a carbazole moiety does not influence the reactivity of the radicals produced (no or weak effect on the acyl radical).

In order to see if these CPO compounds are efficient for the initiation of a polymerization at 395 nm, photopolymerizations were performed with a benchmark trifunctional acrylate monomer, the trimethylolpropane triacrylate (TMPTA). These experiments were performed under air with a low viscous monomer in a thin film, enhancing the oxygen inhibition.

**Figure 4:**
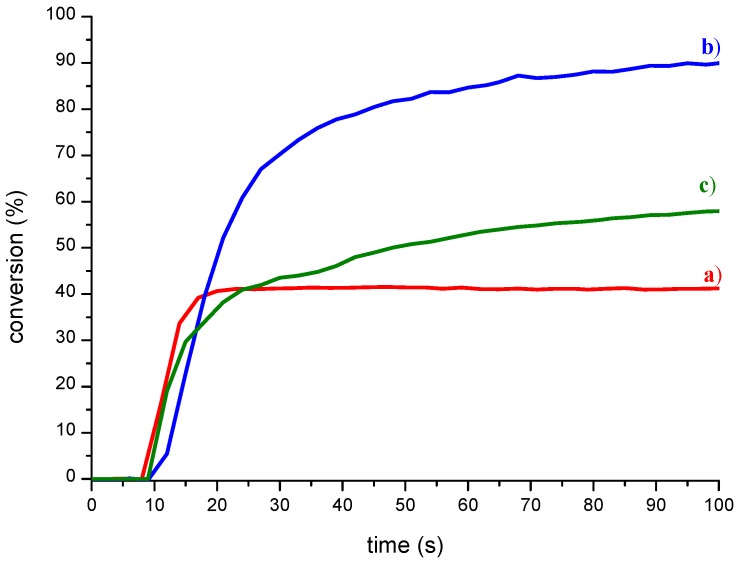
Kinetics of photopolymerization under air (thin layer 25 µm) at 395 nm for TMPTA formulations containing (**a**) 4 wt% CPO-1, (**b**) 4 wt% CPO-2, and (**c**) 4 wt% TPO-L.

Figure 4 shows the photopolymerizations using CPO-1 and CPO-2 compared to TPO-L; this later compound was selected as a reference as it is fully soluble in acrylates for high PI content. A high content of photoinitiator was used to show that i) they can be soluble in acrylates contrary to BAPO (i.e., it was not possible to study BAPO at 4 wt% in TMPTA as it was not fully soluble) and ii) they can initiate the polymerization under air (to overcome the well-established oxygen inhibition in a thin layer and for low viscosity monomers [1]). Not being fully soluble is the main drawback of the use of BAPO when high concentrations are required. Remarkably, the new proposed compounds CPO-1 and CPO-2 were much more soluble in TMPTA than is BAPO. The speed of polymerization was very close for TPO-L, CPO-1, and CPO-2; but the final conversion was much better for CPO-2 with an increase of 50% of the final conversion. Unfortunately, this was not the case for CPO-1, the molecule used for a two-photon initiation. This result shows clearly that the enhanced absorption and the increased cleavage properties of the PI can help to find new PIs that are more efficient than the commercially available TPO-L, even if the phosphinoyl radical was less reactive for the new compound CPO-2. A more refined comparison is not possible as the polymerization is carried out under air for application purposes. This could lead to an increase of reactivity when it is required or to the reduction of the quantity of product needed to reach the same reactivity. This could be an asset from a toxicology point of view, to use as little compound as possible. As the comparison of reactivity was made for a same weight percent, there was already less molar content of CPO-2 (M = 423 g/mol) than TPO-L (M = 316g/mol), showing the enhanced reactivity of the new compound compared to the benchmark photoinitiator. For the CPO initiators, good photobleaching properties were observed with formation of colorless polymers (in the experiments of Figure 4).

## 4. Materials and Methods 

### 4.1. Syntheses of the ADPO Derivatives

All reagents were purchased from Aldrich (Darmstadt, Germany) or TCI-europe (Bruxelles, Belgium) and used as received without further purification. The synthesis is a two-step process. The reactions were performed under inert atmosphere. The second step was carried out in the dark to avoid any reaction with ambient light. 

^1^H and ^31^P NMR spectra were recorded at room temperature on a Varian Oxford 300 spectrometer (Santa Clara, CA, USA): the ^1^H chemical shifts are referenced to the solvent peak CDCl_3_ (7.26 ppm), “br s” is assigned to broad singlets.

#### 4.1.1. Synthesis of Compound CPO-1

1st step: Diphenylphosphine oxide (0.199 g, 0.985 mmol, 1.1 eq.), 9-Ethyl-3-carbazolecarboxaldehyde (0.2 g, 0.896 mmol, 1 eq.), and sodium carbonate (0.104 g, 0.985 mmol, 1.1 eq.) were placed under nitrogen into a dry 5 mL round-bottom flask with a magnetic bar sealed with a septum. After blending, the reaction mixture was heated at 100 °C for 30 min then at 80 °C for 5 hours to obtain a white solid (0.380 g, 100% yield). 

^1^H-NMR (300 MHz, CDCl_3_) δ (ppm): 7.92–7.15 (m, 16H), 6.65 (d, 1H), 4.25 (q, 2H), 1.39 (tr, 3H).

2nd step: The resulting white solid (0.380 g, 0.896 mmol, 1 eq.) was afterward placed into a 50 mL round-bottom flask with MnO2 (1.56 g, 17.9 mmol, 20 eq.) and dichloromethane (50 mL). The mixture was stirred under nitrogen for 22–24 h at 90 °C in the dark. The mixture was filtered over celite and rinsed 3 times with dichloromethane. The solvent was evaporated under reduced pressure. An off white powder was obtained (380 mg, 100% yield).

^1^H-NMR (300 MHz, CDCl_3_) δ (ppm): 8.67 (d, 1H), 8.15 (d, 1H), 8.02–7.12 (m, 15H), 4.19–4.00 (m, 2H), 1.47–1.27 (m, 3H).

^31^P NMR (162 MHz, CDCl_3_) δ (ppm): 22.5.

#### 4.1.2. Synthesis of Compound CPO-2

1st step: Diphenylphosphine oxide (0.15 g, 0.77 mmol, 1.1 eq.), 9-benzylcarbazole-3-carboxaldehyde (0.2 g, 0.7 mmol, 1 eq.), and sodium carbonate (0.08 g, 0.77 mmol, 1.1 eq.) were placed under nitrogen into a dry 5 mL round-bottom flask with a magnetic bar sealed with a septum. After blending, the reaction mixture was heated at 120 °C for 1 h then 100 °C for 5 h to obtain a yellow solid (0.34 g, 89% yield).

^1^H NMR (300 MHz, CDCl_3_) δ (ppm): 7.94–7.29 (m, 16H), 7.24–7.16 (m, 4H), 7.10–7.07 (m, 2H), 5.67 (s, 1H), 5.47 (s, 2H)

2nd step: The resulting yellow solid (0.34 g, 0.69 mmol, 1 eq.) was afterwards placed into a 100 mL round-bottom flask with MnO_2_ (1.2 g, 13.98 mmol, 20 eq.) and dichloromethane (40 mL). The mixture was stirred under nitrogen for 22–24 h at room temperature in the dark and then filtered over celite. The latter product was rinsed 3 times with dichloromethane. The filtrate was concentrated under reduced pressure to give a yellow gum (3.94 g, 99% yield).

^1^H NMR (300 MHz, CDCl_3_) δ (ppm): 8.64 (d, 1H), 8.22 (d, 1H), 7.98–7.91 (m, 4H), 7.58–7.30 (m, 10H), 7.28–7.23 (4H), 7.13–7.10 (m, 2H), 6.53 (s, 2H).

^31^P NMR (162 MHz, CDCl_3_) δ (ppm): 22.38.

### 4.2. Other Chemicals

Trimethylolpropane triacrylate (TMPTA) was provided by Allnex (Bruxelles, Belgium). BAPO and TPO-L were a gift from BASF (Ludwigshafen, Germany). Acetonitrile was purchased from Fluka (Strasbourg, France). 

### 4.3. UV-Vis Spectroscopy

#### 4.3.1. Spectrum Recording

UV-visible spectra were recorded in acetonitrile in a quartz cell on a Varian® Cary 3 spectrometer.

#### 4.3.2. Photolysis Experiment

The quantum yields of photolysis Φ were measured experimentally using BAPO as a reference (Φ_BAPO_ = 0.6) using the procedure described in [22]. A 4 mL of solution of PI in acetonitrile was stirred under nitrogen and irradiated with a LED at 385 nm (48 mW/cm²). Absorption spectra were recorded at different times during the irradiation.

The following equation (equation 1 below) was used to compute the quantum yield of photolysis for the new PI [22]:

(1) $$\Phi_{\mathrm{sample}}=\Phi_{\mathrm{BAPO}}.\left[\varepsilon_{\mathrm{BAPO}}\cdot {\mathrm{slope}}_{\mathrm{sample}}\right]/\left[\varepsilon_{\mathrm{sample}}\cdot {\;\mathrm{slope}}_{\mathrm{BAPO}}\right]$$

where slope is defined as the slope of the function ln[exp(2,3.OD_385 nm_) − 1] = f(t) and OD is the optical density or absorbance in the function at time (t).

### 4.4. Molecular Modeling

#### 4.4.1. Geometries Optimization

Geometry optimizations [26] were calculated at an UB3LYP/6-31G* level of theory, and geometries were frequency checked (Gaussian 09 software). 

#### 4.4.2. UV-Vis Spectrum Calculation

The electronic absorption spectra were calculated from the time-dependent density functional theory at MPW1PW91/6-31G* level of theory on the relaxed geometries calculated at the UB3LYP6-31G* level of theory

### 4.5. RT-FTIR Spectroscopy

A Jasco 6600 Real-Time Fourier Transformed Infrared Spectrometer (RT-FTIR) (Tokyo, Japan) was used to follow the C=C double bond conversion versus time for polymerizations of 25 µm thick samples (the photosensitive resin was spread on the surface of a BaF2 pellet for RT-FTIR investigation). All the photopolymerization experiments were carried out at room temperature (RT) (21−25 °C) under air (oxygen inhibition expected [22]).

The evolution of the infrared acrylate C=C double bond peak was followed around 1650 cm^−1^. A light emitting diode LED@395 nm (Thorlabs, Maisons-Laffitte, France) having an irradiance of 77 mW/cm² at the sample position was used for the photopolymerization experiments. The FTIR spectra are followed in real time upon LED irradiation to determine the conversion (from the C=C peak area).

## 5. Conclusions

In this paper, two CPO derivatives were synthesized and studied as type I photoinitiators. As expected, the introduction of a carbazole chromophore enhanced the absorption of these molecules in the 365–405 nm range. The cleavage reaction was not strongly modified but was slightly increased, mostly in CPO-2. The reactivity of CPO-2 is therefore better than that of the commercial photoinitiator TPO-L, showing the potential of this new molecule to be a photoinitiator adapted for LED applications. One of the main advantages would be the lower amount of PI needed in a formulation compared to TPO-L or BAPO to reach the same reactivity, and that could be an asset from a toxicological point of view. The search for new PIs is still underway and uses molecular orbital calculations to help design new PIs.

## Figures and Tables

**Table 1:** Predicted properties: maximum of absorption (λ max), computed by molecular modeling, compared to experimental findings; molar extinction coefficient (ε_395 nm_) measured in acetonitrile at 395 nm, and quantum yield of photolysis (ϕ) in acetonitrile.

	Molecular Modeling	Experimental Data	
Molecules	λ max (nm) (Oscillator Strength)	ε_395 nm_ (L/mol/cm)	ϕ
TPO-L	383 (0.0005)	131	0,3 [5]
BAPO	384 (0.0006)	664	0,6 [22]
CPO-1	384 (0.007)	5600	0,7
CPO-2	385 (0.007)	3200	0,8
